# Case Report: A 3D-Printed Surgical Guide for Breast-Conserving Surgery After Neoadjuvant Chemotherapy

**DOI:** 10.3389/fonc.2021.633302

**Published:** 2021-03-25

**Authors:** Zhen-Yu Wu, Guk Bae Kim, Sangwook Lee, Seung Hyun Choi, Namkug Kim, BeomSeok Ko

**Affiliations:** ^1^ Department of Breast Surgery, Shanghai East Hospital, Tongji University School of Medicine, Shanghai, China; ^2^ Division of Breast Surgery, Department of Surgery, Asan Medical Center, University of Ulsan College of Medicine, Seoul, South Korea; ^3^ Biomedical Engineering Research Center, Asan Institute for Life Sciences, Asan Medical Center, Seoul, South Korea; ^4^ Research Department, Anymedi Inc., Seoul, South Korea; ^5^ Department of Radiology, Asan Medical Center, University of Ulsan College of Medicine, Seoul, South Korea

**Keywords:** breast-conserving surgery, breast surgical guide, neoadjuvant chemotherapy, three-dimensional-printing, tumor localization

## Abstract

**Background:**

A challenging problem for patients undergoing breast-conserving surgery after neoadjuvant chemotherapy (NACT) is the accuracy of preoperative tumor localization. After chemotherapy, the original tumor is likely to shrink or scatter dramatically or even show complete remission. For breast-conserving surgery, the development of a guidance device to accurately estimate the resection area is imperative.

**Case Presentation:**

We produced a three-dimensional (3D)–printed breast surgical guide (BSG) based on prone and supine magnetic resonance imaging (MRI). This device was tested on a patient who underwent breast-conserving surgery after NACT. Both ultrasonography and MRI revealed that the tumor shrank substantially after NACT. Identifying the target tumor area using pre-NACT MRI was feasible, and the tumor was safely removed with clear resection margins.

**Conclusion:**

The BSG has several advantages over conventional methods for tumor localization after NACT. In particular, the BSG provided precise quantitative MRI information about the tumor area.

## Introduction

Neoadjuvant chemotherapy (NACT) is the standard treatment for locally advanced breast cancer (1, 2). NACT plays an important role in downstaging the extent of the tumor, allowing patients to undergo breast-conserving surgery instead of total mastectomy (3). However, accurate preoperative tumor localization after NACT is challenging; the original tumor is likely to shrink or scatter dramatically or even undergo complete remission after chemotherapy. Development of a guidance device that facilitates the accurate estimation of the resection area for breast-conserving surgery after NACT is imperative. In a previous pilot study conducted at our institution (4), the authors described the clinical feasibility of a prone magnetic resonance imaging (MRI)–based three-dimensional (3D)-printed breast surgical guide (BSG) for breast-conserving surgery after NACT. The BSG can project the pre-NACT tumor area on the affected breast (4). However, the shape and location of the breast and tumor differ between the prone position and the supine position generally adopted for surgery. To improve the accuracy of tumor localization, we applied a prone and supine MRI-based 3D-printed BSG for precise breast-conserving surgery in a patient who had received NACT for breast cancer.

## Case Presentation

### Clinical History

**Figure 1:**
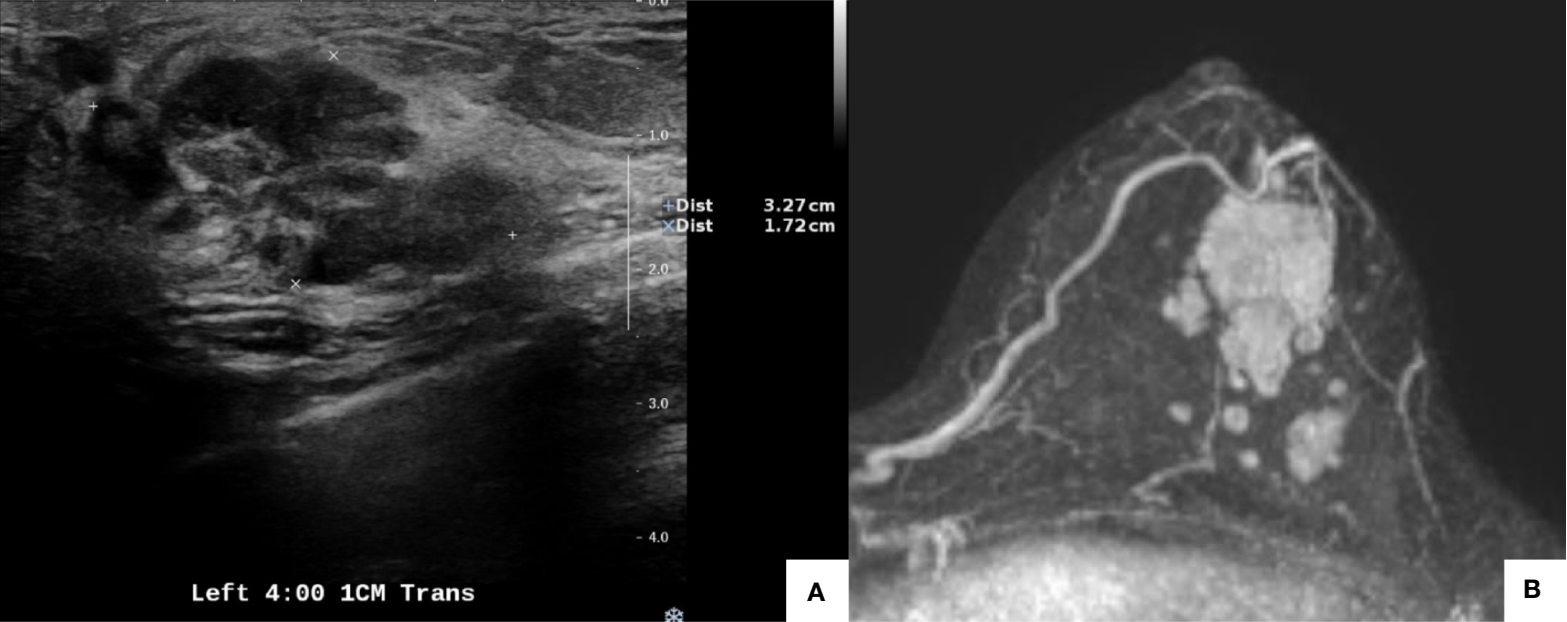
Pretreatment imaging evaluation. Ultrasonography showed an irregular hypoechoic mass at the 4-o’clock position, 1 cm from the nipple, measuring 3.9 × 3.3 cm. Malignancy was confirmed with a biopsy **(A)**. Magnetic resonance imaging revealed a mass (3.4 × 2.2 cm) at the 4-o’clock position, 1 cm from the nipple in the left breast, which was concordant with the biopsy-confirmed malignancy. Multiple enhanced nodules (1.4 cm in the longest diameter) were observed, and the total extent measured 5.8 cm **(B)**.

A 38-year-old woman was diagnosed with invasive breast cancer at her local hospital. The patient was referred to our department in February 2020. Physical examination revealed a well-defined palpable mass at the 3–4-o’clock position of her left breast. Ultrasonography revealed an irregular hypoechoic mass at the 4-o’clock position, 1 cm from the nipple, measuring 3.9 × 3.3 cm, which was consistent with the biopsy-confirmed malignancy (**Figure 1A**). Two hypoechoic suspicious daughter nodules in the 3:30 direction, 5 cm and 4 cm from the nipple, with diameters of 1.2 cm and 0.6 cm, respectively, were also detected. Additionally, multiple enlarged lymph nodes suspicious for metastases were detected in the left axilla, level I to III.

A core needle biopsy of the left-sided 3-o’clock mass confirmed invasive ductal carcinoma, of nuclear grade (NG) 2/3 and histologic grade (HG) 2/3, which was estrogen receptor (ER) positive (6/8), progesterone receptor (PR) positive (6/8), and human epidermal growth factor receptor 2 (HER2) negative (1+), with a Ki-67 proliferation index of 10%–20%. A left axillary lymph node needle aspiration biopsy revealed metastatic carcinoma. A whole-body CT scan revealed no evidence of distant metastasis.

### Neoadjuvant Chemotherapy

The patient’s TNM staging was cT2N1M0. Following discussions, the patient agreed to NACT with four cycles of an anthracycline-based treatment followed by four cycles of a taxane. Pre-NACT enhanced breast MRI revealed a mass (3.4 × 2.2 cm) at the 4-o’clock position, 1 cm from the nipple, in the left breast, which was concordant with the biopsy-confirmed malignancy. Multiple enhanced nodules (1.4 cm in the longest diameter) were observed, and the total extent measured 5.8 cm (**Figure 1B**). MRI revealed multiple enlarged lymph nodes in the left axilla.

**Figure 2:**
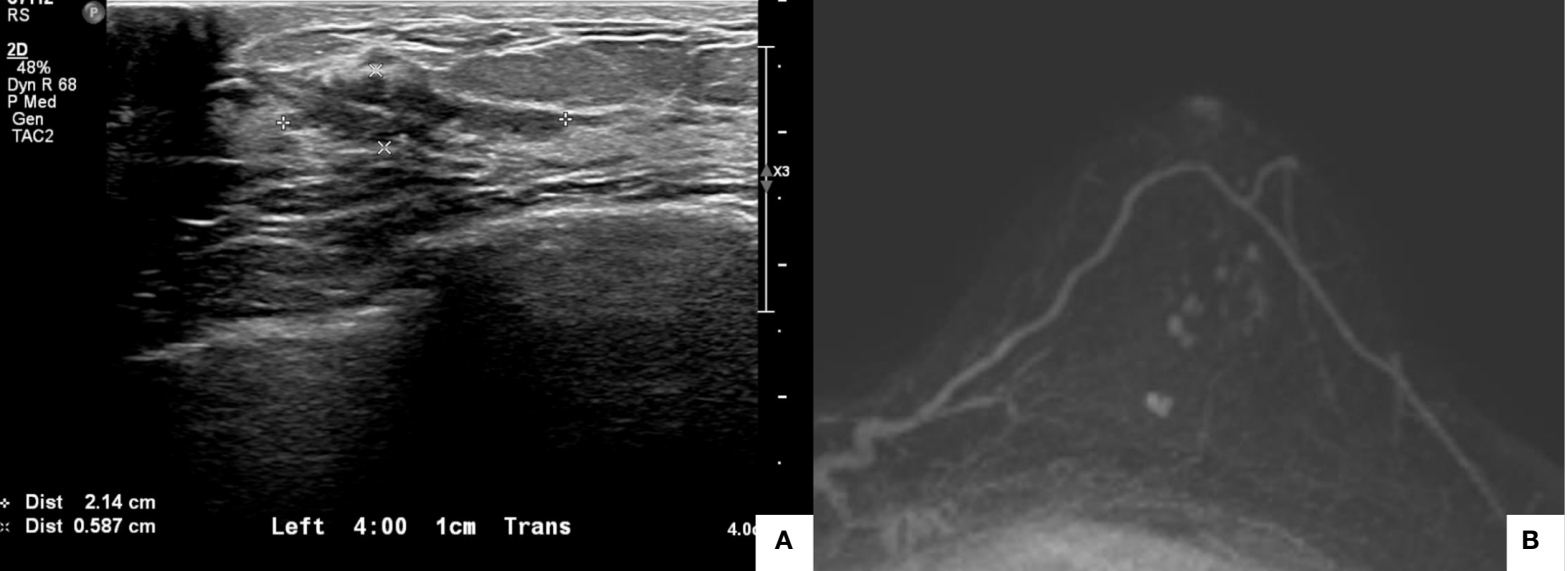
Posttreatment imaging evaluation. Ultrasonography showed that the dimensions of the malignant mass had decreased to 2.1 × 1.0 cm **(A)**. Magnetic resonance imaging showed that the biopsy-confirmed malignant mass and multiple suspicious daughter nodules (total extent measuring 2.1 cm) in the left breast had decreased in size **(B)**.

The patient received four cycles of NACT with 60 mg/m^2^ of intravenous adriamycin and 600 mg/m^2^ of intravenous cyclophosphamide, plus 75 mg/m^2^ of intravenous docetaxel. After the fourth cycle, the dimensions of the malignant mass had decreased to 2.1 × 1.0 cm, as revealed by ultrasonography of the left breast (**Figure 2A**). The two daughter nodules in the 3:30 direction shrank to 0.5 cm and 0.4 cm. The left-axillary lymph nodes (levels I to III) shrank slightly. The post-NACT MRI showed that the biopsy-confirmed malignant mass and the multiple suspicious daughter nodules (total extent measuring 2.1 cm) in the left breast also decreased in size (**Figure 2B**).

### Magnetic Resonance Imaging-Based 3D-Printed Surgical Guide

**Figure 3:**
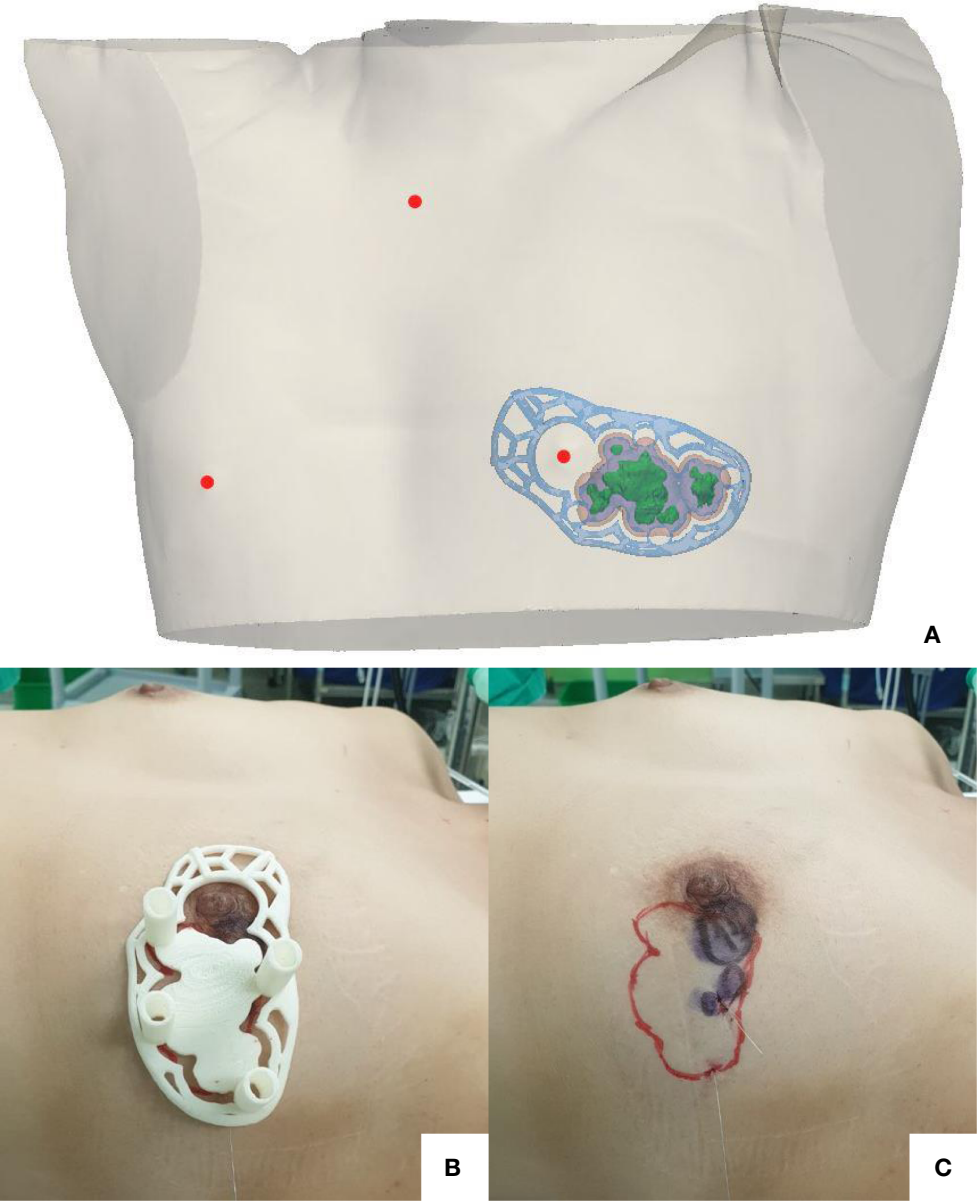
The breast surgical guide (BSG) targeted the original tumor region by combining pretreatment prone magnetic resonance imaging (MRI)-generated tumor data and posttreatment supine MRI-generated breast data **(A)**. The BSG was manufactured as a hybrid type, containing a groove for marking the original tumor area on the breast surface with an extra 0.5 cm from the tumor boundary to guarantee safe margins. Blue dye injection columns were provided to indicate the extent of the tumor requiring removal **(B)**. A surgical resection line was drawn onto the breast skin surface along with a groove designed to match the original tumor area **(C)**.

We proposed breast-conserving surgery using a prone/supine MRI-based 3D-printed BSG to facilitate the excision of the main breast tumor along with the suspicious daughter nodules. Breast imaging was performed using a 3.0 T MRI system (Ingenia; Philips Healthcare, Best, the Netherlands) with a bilateral dedicated 4-element breast coil. Additional supine MRI was performed to replicate the patient’s intraoperative position. The patient provided written informed consent and agreed to undergo supine imaging in addition to the standard baseline MRI protocol. Data obtained from the prone/supine MRI scans were analyzed. The tumor and normal breast tissues were divided using the image segmentation program Mimics Medical v17 (Materialise Inc., Leuven, Belgium). We designed a BSG to target the original tumor region by combining pre-NACT MRI-generated tumor data with post-NACT MRI-generated breast data (**Figure 3A**). The 3D-printed BSG was designed not to target the tumor region depicted in pre-NACT magnetic resonance (MR) images but to mark the reduced tumor area by comparing pre-NACT MR images with post-NACT MR images. To ensure an accurate projection of the tumor resection boundary, the following specifications were used for modeling the BSG: (1) The BSG was designed to fit precisely on the breast skin surface. (2) A hole was made to fit the nipple and prevent rotation of the BSG; guidelines indicating the contralateral nipple and the suprasternal notch were included on the BSG. (3) The BSG was manufactured as a hybrid type with a groove for marking the original tumor area with an additional 0.5 cm around the tumor boundary to guarantee safe margins. Blue dye injection columns were incorporated to indicate the extent of the tumor requiring removal.

### Breast-Conserving Surgery With the 3D-Printed Surgical Guide

The surgery was performed in August 2020. The 3D-printed BSG was sterilized preoperatively. The surgical resection line was drawn onto the breast skin surface along with the groove designed to match the tumor shape, and blue dye was injected into the breast through the columns indicating the resection boundary (**Figures 3B, C**). A left-sided lumpectomy and sentinel lymph node biopsy were performed. The tumor was removed using the blue dye border as the excision boundary. An intraoperative frozen biopsy of the resection margins yielded negative results. Sentinel lymph node frozen biopsies revealed one of five lymph nodes positive for metastatic carcinoma, and axillary lymph node dissection was performed. The total operation time was 81 minutes.

### Pathological Results and Adjuvant Therapy

The final histopathologic diagnosis of the specimen was residual invasive lobular carcinoma: NG 3/3, HG 2/3, ypT2 (3.2 cm) N1 (2/12) M0, stage IIB, ER (+, 34-66%), PR (+, 11-33%), HER2 (1+), and Ki-67 <10%. All resection margins were free of tumor cells. The distance from the tumor to the resection margin was 3.2 cm, 2 cm, 1.2 cm, and 1 cm in the 3-, 6-, 9-, and 12- o’clock directions, respectively. The mean distance from the tumor to the resection margin was 1.9 cm. The patient underwent adjuvant radiotherapy and hormonal therapy with tamoxifen. At the last follow-up before this report, the patient was in a good condition.

## Discussion

In a meta-analysis including 10 randomized controlled trials, the long-term survival associated with NACT was equivalent to that associated with adjuvant chemotherapy. However, NACT was associated with more local recurrences than adjuvant chemotherapy among patients who underwent breast conservation therapy (5). This result highlights the importance of an accurate tumor localization strategy for patients undergoing breast-conserving surgery after NACT (5). Substantial remission of the original tumor usually occurs following NACT, making accurate tumor localization difficult.

Conventional methods, such as hook-wire localization, clip marker insertion, and radioactive seeding techniques, have been used to localize breast tumors (6–9). However, these localization methods do not accurately project the initial tumor bed after a substantial remission following NACT (6–9). Additionally, each localization technique has its inherent shortcomings and limitations. For example, hook-wire–guided localization, the most commonly used method, causes pain and complications, such as bleeding and pneumothorax. Moreover, hook-wires are occasionally cut off or lost during localization procedures (8). Clip markers are inserted at the tumor site during the NACT course to mark the original tumor bed; however, this technique is associated with pain and bleeding, and the inserted clip marker is sometimes difficult to find during surgery because it can migrate from the initial location (7, 8). Radioactive seeding localizations require strict nuclear regulatory protocols for storage, access, monitoring, and disposal of the radioactive seeds, which may complicate workflow and logistic considerations (8, 9).

The aforementioned localization methods are generally performed under ultrasonographic or mammographic guidance in current clinical practice. The sensitivity of MRI at predicting tumor extent after NACT has been reported to be superior to other imaging modalities (10). Considering the limitations of conventional localizations, an MRI-based 3D-printed BSG can be an effective alternative. First, a 3D-printed BSG can precisely project the original tumor extent in the affected breast using pre- and post-NACT MRI information. This precision is particularly useful when managing patients with multiple or fragmented tumors that have responded well to NACT. Second, compared with conventional methods, such as hook-wire localization or radioactive seeding, a 3D-printed BSG is less invasive and more patient-friendly, as it is not associated with pain or radiation exposure. Third, the hook-wire method requires a radiology team to perform the wire insertion at a specified time, and radioactive seeding requires regulatory protocols. However, the production of a BSG is based on the patient’s MRI information; thus, the workflow is simpler and more flexible.

In a previous pilot study conducted at our institution, the authors employed prone MRI-based 3D-printed BSGs for breast-conserving surgery on patients who had received NACT (4). The BSG was customized using pre- and post-NACT MRI data obtained in the prone position. The preliminary data demonstrated the clinical feasibility and effectiveness of this novel technology (4). However, a BSG based on MRI data obtained with the patient in the supine position may be more appropriate because the surgery is generally performed with the patient supine. Thus, MRI in the supine position should increase the accuracy of the BSG, leading to better clinical outcomes. For the patient reported herein, we combined prone and supine MRI data when manufacturing the BSG to improve the accuracy of the tumor localization. The tumor shrank substantially after NACT, as revealed by both ultrasonography and MRI. However, identifying the target tumor area using the pre-NACT MRI alone was feasible. The purpose of a 3D-printed BSG is not to target the tumor area delineated by pre-NACT MRI but to mark the reduced tumor range by comparing pre- with post-NACT MRI. Moreover, through the columns that were modeled in three dimensions in the BSG, the blue dye injection into the breast tissue provided additional quantitative representation of the resection boundary. The tumor was successfully removed with clear resection margins using the device.

In conclusion, we applied a prone and supine MRI-based 3D-printed BSG for a patient undergoing breast-conserving surgery after NACT, and the tumor was safely removed. BSGs have several advantages over conventional methods for tumor localization in the NACT setting. In particular, BSGs provide quantitative information from MRI about the original tumor area before treatment. In the future, the applicability of MRI-based 3D-printed BSGs for patients with occult breast lesions or non-palpable breast cancer deserves further exploration. Additionally, evaluations of aesthetic results, cost-effectiveness, and patient quality of life following this technique, in comparison with conventional localization methods, are needed.

## Data Availability Statement

The raw data supporting the conclusions of this article will be made available by the authors, without undue reservation.

## Ethics Statement

The studies involving human participants were reviewed and approved by Asan Medical Center. The patients/participants provided their written informed consent to participate in this study. Written informed consent was obtained from the individual(s) for the publication of any potentially identifiable images or data included in this article.

## Author Contributions

BK designed and conducted the research. Z-YW and BK performed the data acquisition and analysis. Z-YW wrote the main manuscript text. GBK, SL, SHC, and NK developed methods and help this experiment. All authors contributed to the article and approved the submitted version.

## Funding

This work was supported by the Ministry of Trade, Industry & Energy (MOTIE), Korea Institute for Advancement of Technology (KIAT), through the Industrial Technology Innovation Program, P0008801.

## Conflict of Interest

Authors GK, SL, and SC were employed by company Anymedi Inc. Z-YW received consulting fees from the company Anymedi Inc. NK is a stakeholder of Anymedi Inc.

The remaining authors declare that the research was conducted in the absence of any commercial or financial relationships that could be construed as a potential conflict of interest.
